# Maxwell’s Demon in Quantum Mechanics

**DOI:** 10.3390/e22030269

**Published:** 2020-02-27

**Authors:** Orly Shenker, Meir Hemmo

**Affiliations:** 1 Department of Philosophy, The Hebrew University of Jerusalem Mount Scopus, Jerusalem 91905, Israel; 2 Department of Philosophy, University of Haifa, Haifa 31905, Israel; meir@research.haifa.ac.il

**Keywords:** entropy, Maxwell’s Demon, the second law of thermodynamics, arrow of time

## Abstract

Maxwell’s Demon is a thought experiment devised by J. C. Maxwell in 1867 in order to show that the Second Law of thermodynamics is not universal, since it has a counter-example. Since the Second Law is taken by many to provide an arrow of time, the threat to its universality threatens the account of temporal directionality as well. Various attempts to “exorcise” the Demon, by proving that it is impossible for one reason or another, have been made throughout the years, but none of them were successful. We have shown (in a number of publications) by a general state-space argument that Maxwell’s Demon is compatible with classical mechanics, and that the most recent solutions, based on Landauer’s thesis, are not general. In this paper we demonstrate that Maxwell’s Demon is also compatible with quantum mechanics. We do so by analyzing a particular (but highly idealized) experimental setup and proving that it violates the Second Law. Our discussion is in the framework of standard quantum mechanics; we give two separate arguments in the framework of quantum mechanics with and without the projection postulate. We address in our analysis the connection between measurement and erasure interactions and we show how these notions are applicable in the microscopic quantum mechanical structure. We discuss what might be the quantum mechanical counterpart of the classical notion of “macrostates”, thus explaining why our Quantum Demon setup works not only at the micro level but also at the macro level, properly understood. One implication of our analysis is that the Second Law cannot provide a universal lawlike basis for an account of the arrow of time; this account has to be sought elsewhere.

## 1. Introduction

J. C. Maxwell devised his so-called Demon in 1867 to show that the Second Law of thermodynamics cannot be universally true if classical mechanics is universally true. In this paper we show that a Maxwellian Demon is compatible with standard quantum mechanics.

Maxwell’s Demon is a way of demonstrating that the laws of mechanics are compatible with microstates and Hamiltonians that lead to an evolution which violates the Second Law of thermodynamics by transferring heat from a cold gas to a hot one without investing work. That is, the Demon is not a practical proposal for constructing a device that would violate the Second Law of thermodynamics, but rather a statement to the effect that the Second Law cannot be universally true if the laws of mechanics are universally true.

Attempts to prove the Second Law as a universal theorem in classical statistical mechanics, encounter the following issue: since the fundamental microscopic dynamics is invariant under velocity reversal, one can show that given any Hamiltonian, if there are entropy-increasing trajectory segments in the system’s state space, then there are also entropy-decreasing ones, and so the thermodynamic law cannot be strictly true. Essentially the same issue arises in the context of the standard approaches to quantum statistical mechanics (see [1,2,3,4,5,6]). The prevalent approaches for explaining why we do not encounter anti-thermodynamic evolutions is that those are extremely a-typical or unlikely. The meaning of typicality and probability in this context is a subject of ongoing debates in both classical and quantum statistical mechanics. The standard approaches, however, depend on a choice of measure on the state space, which in our view is not dictated by the underlying theory (see [7,8]). For other views and the debate on the meaning of typicality and the difference between typicality and probability, see [9,10,11,12,13,14,15,16]. In the context of the question of the Demon, one needs to distinguish between the Second Law and the law of approach to equilibrium, which underlies it; see [17].

Since Maxwell, many attempts have been made to disprove Maxwell’s intuition. In [7,18,19,20,21,22] we provide a phase space argument to demonstrate that a classical mechanical macroscopic evolution that satisfies Liouville’s theorem can be entropy decreasing. By this we have shown that classical mechanics does not rule out a dynamical evolution that takes most or even all the points in an initial macrostate of the universe to a macrostate with a smaller Lebesgue measure, i.e., with lower total entropies. (In Albert’s proof [10] of a Maxwellian Demon in the classical context the thermodynamic cycle is not complete. The missing link to complete the cycle required proving that erasing the Demon’s memory is not dissipative. More generally, one needs to show that the so-called Landauer–Bennett thesis is mistaken. In [7,18,19,20,21,22] we provided this missing link; in [19] we described a concrete example of a Demonic setup in Szilard’s engine).

The situation just described is in the context of classical mechanics. There is a vast literature (see [23] and references therein) on the question of Maxwell’s Demon also in the context of quantum mechanics. Most of the literature is based on the classical Landauer–Bennett thesis (see [24,25,26,27,28]; for the use of the classical thesis in the quantum context, see the discussion by Earman and Norton [29] of Zurek’s [30]), which is central in the contemporary attempts to exorcise Maxwell’s Demon. 

In the literature about the Landauer–Benett thesis, the notion of “entropy” is usually applied without explaining it. However, as is well known, there are two “theoretical frameworks”, both called statistical mechanics, that offer two different notions of entropy that are supposed to account (at least approximately) for the thermodynamic notion of entropy (See [10,31,32,33,34,35,36]: one framework follows the work of Boltzmann and the other of Gibbs. The notion standardly used in the literature in classical statistical mechanics is a Boltzmannian one: the entropy of a system in a given microstate is a function of the (Lebesgue) measure of the macrostate to which this microstate belongs. The Gibbs entropy is defined for systems in equilibrium, where “equilibrium” is understood as a measure that is invariant under the dynamics. On this account, “entropy” is a function of the entire phase space, that may be seen as some sort of weighted averages calculated given the appropriate measures over that space (for the appropriate ensembles). A consequence of this is that this notion of Gibbsian entropy remains constant, and cannot account for the approach to thermodynamic equilibrium. To solve this problem Gibbs introduced the idea of successive coarse graining. Unlike the Boltzmannian coarse graining into macrostates, the Gibbsian coarse graining is not associated with macrovariables, and moreover, it needs to change constantly in a way that makes the graining finer and finer, and in the limit the graining has to be maximally finer (e.g., finer than the capabilities of any measuring device). The Gibbsian approach is known to be conceptually very problematic; see [37,38]). One explanation for its usefulness in practice is that it can be explained in terms of Botlzmann’s macrostates, if the dynamics is taken into account; this idea is described in [7], Chapter 11). It seems to us that the Gibbsian notion of entropy cannot be usefully applied in the context of Landauer’s thesis, which concerns the evolution of a system from an initial macrovariable to a final one. In our view, the “translation” of this idea to Gibbsian terms leaves out the essential magnitudes of this thesis, and the arguments for it; see [38].

In his ground-breaking paper, Landauer [24] described his thesis in the classical context as follows.

“Consider a statistical ensemble of bits in thermal equilibrium. If these are all reset to one, the number of states covered in the ensemble has been cut in half. The entropy therefore has been reduced by klog2 = 0.6931 per bit. The entropy of a closed system, e.g., a computer with its own batteries, cannot decrease; hence this entropy must appear elsewhere as a heating effect, supplying 0.6931 kT per restored bit to the surroundings.”

(See [24], p. 265)

According to this line of thinking, the thesis is grounded in the Second Law of thermodynamics (or its statistical mechanical counterparts). This is the prevalent approach, both in arguments supporting Landauer’s thesis (including e.g., [26,27,39,40,41,42,43]) and in arguments criticizing it (including e.g., [29,39,44,45,46]). (The connection between information, entropy and probability is also under dispute: see [47,48]).

One problem in grounding Landauer’s thesis in the Second Law is that Landauer’s thesis is itself central in contemporary defenses of the universality of the Second Law; thus, relying on the Second Law to establish Landauer’s thesis is viciously circular. There are two ways to establish the universal truth of the Second Law of thermodynamics. One is on the basis of empirical evidence: the Second Law enjoys enormous empirical support, and the overwhelming empirical evidence makes it uncontroversial that there are no perpetual motion machines in our world. (Recent attempts to test experimentally Landauer’s thesis are, e.g., [49,50,51]). The second way to establish the universal truth of the Second Law is by showing that this universal truth is a theorem of fundamental physics (which is, in turn, taken to be fundamentally universally true). Maxwell’s Demon is a thought experiment that challenges this latter grounding of the Second Law.

Importantly, Maxwell’s Demon is not in conflict with the empirical evidence, because the available proofs that Maxwell’s Demon is compatible with fundamental physics leave open the possibility that both Second Law behavior and Maxwellian Demon behavior are compatible with fundamental physics; they may hold for different initial conditions of the universe, for example. Nevertheless, for many thinkers, this last option is not satisfactory, and they strive to prove that Maxwellian Demons are incompatible with fundamental physics. One way of doing so, in fact the most prevalent way in contemporary research, is by relying on Landauer’s thesis (see [23] and references therein). For this reason, relying on the Second Law in establishing Landauer’s thesis is circular if that law itself is defended by relying on Landauer’s thesis. Not every circularity is vicious; it seems to us that this one is vicious (see also [29,44]). In order to defend the universality of the Second Law, Landauer’s thesis should be grounded, not in the Second Law, but in independent arguments based on fundamental physics. (We thank James Ladyman for a correspondence about this point).

The same idea applies to the question of Maxwell’s Demon, which can be phrased roughly as follows: Can there be, as a matter of principle, a mechanical system (even highly idealized), and dynamical evolution that satisfy the laws of mechanics (quantum or classical), but violate the Second Law of thermodynamics? Our answer in this paper is in the affirmative.

We consider a thought experiment along the lines of Szilard’s (see [52]) and Bennett’s (see [26,27]) particle-in-a-box, in a quantum mechanical context (see our [19] for a classical analysis of this setup). Since we are interested in the question of whether or not thermodynamics is consistent with quantum mechanics as a matter of principle, we consider the experiment in a highly idealized framework, disregarding practical questions (some of which we shall mention along the way). (We do not address the question of whether a single particle in a box is a thermodynamic system; see e.g., [53]). Since the arguments in the literature concerning the entropic cost of measurement and erasure have been given in this setup, this is the setup we consider). In particular we assume, for simplicity only, that in our idealized setup there is no environmental decoherence. (For the role of the number of degrees of freedom in macroscopic irreversibility, see [54]). For quantum decoherence theory and its role in recovering the so-called classical limit of quantum mechanics, see [55]). We explain in the last section why our argument is (in principle) applicable also in the presence of decoherence.

Our discussion is in the framework of standard quantum mechanics in von Neumann’s 1932 formulation (see [56]). We give two separate arguments in the context of standard quantum mechanics: one argument is in the context of quantum mechanics with the so-called projection postulate, and another without it. We set aside questions concerning the physical interpretation of such theories, nor do we consider realistic applications of our setup.

The structure of the paper is this. In Section 2 we start with an analysis of a Demonic evolution in standard quantum mechanics without the projection postulate. In Section 3 we consider the same setup in quantum mechanics with the projection postulate. In Section 4 we describe the connection between measurement and erasure in quantum mechanics with and without the projection postulate. We conclude in Section 5 with some remarks addressing decoherence and the question of whether our argument for a quantum mechanical Maxwellian Demon can be carried over to a macroscopic setup in quantum statistical mechanics. In Appendix A we describe briefly the notion of a quantum macrostate.

## 2. A Quantum Mechanical Demon in Unitary Quantum Mechanics

**Figure 1:**
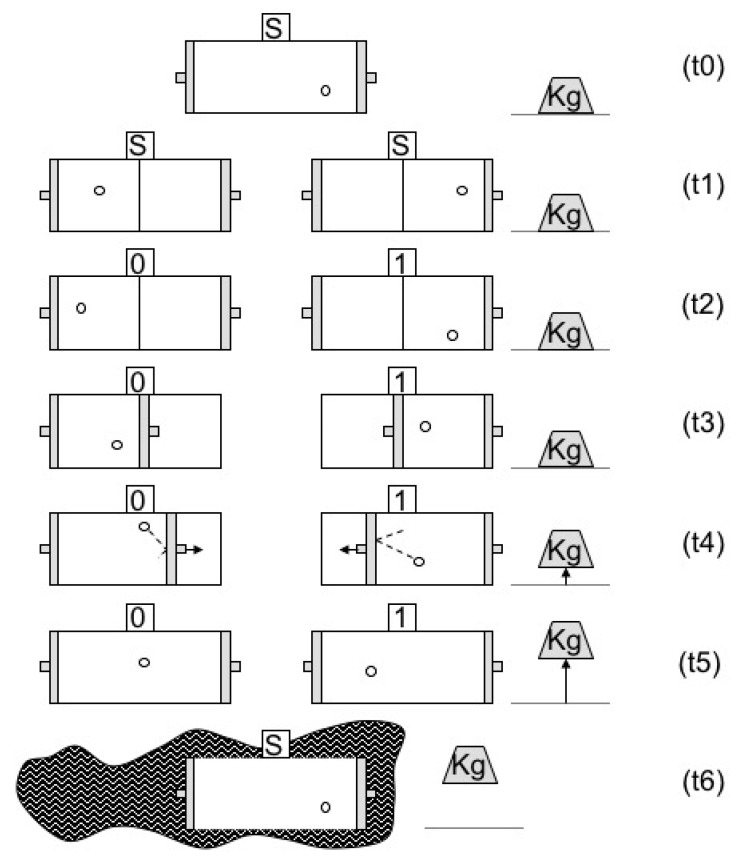
A quantum mechanical Maxwellian Demon.

Consider the setup in Figure 1. At $t_{0}$ a particle is placed in a box. At $t_{1}$ a partition is inserted exactly at the center of the box so that the particle is trapped in the left-hand side L or the right-hand side R of the box. At $t_{2}$ a measurement of the location of the particle, left or right, is carried out and the outcome of the measurement, 0 or 1, respectively, is registered in the memory state of the measuring device. At $t_{3}$ the partition is replaced by a piston (in accordance with the measurement outcome), which is subsequently pushed by the particle at $t_{4}$. The piston is coupled to a weight located outside the box which is raised during the expansion. At $t_{5}$ the particle is again free to move throughout the box and the weight is at its maximal height. At $t_{6}$ the memory of the device is erased and returns to its initial standard state. The particle returns to its initial energy state by receiving from the environment the energy it has lost to the weight. The cycle of operation is thus closed. By this last statement we mean that everything returns to its initial state except for the energy transfer from the heat bath (environment) to the weight (see our [7,18,19]).

We will now show that according to quantum mechanics this setup can be a Maxwellian Demon. We start by constructing a quantum mechanical microscopic dynamical evolution that satisfies the Schrödinger equation at all times (i.e., without applying the projection postulate in measurement). We consider the implications of our setup in standard quantum mechanics with the projection postulate in the next section. In both cases, our dynamical evolution includes measurement and erasure under idealized assumptions. Again, we set aside questions concerning the interpretation of such theories. In the last section we argue that as a matter of principle from our microscopic setup one can construct a quantum mechanical macroscopic Demonic evolution. We discuss in Appendix A the notion of the quantum mechanical analogue of a classical macrostate, according to which the system is in a microstate, but there is ignorance concerning which quantum microstate it is.

At $t_{0}$ the quantum state of the entire setup is the following:

(1) $$|\Psi (0)\rangle ={|x_{0}\rangle }_{p}{|S\rangle }_{m}{|down\rangle }_{w}{|e_{0}\rangle }_{e}$$

where ${|x_{0}\rangle }_{p}$ is the initial state of the particle in a one-dimensional box; ${|S\rangle }_{m}$ is the initial standard (ready) state of the measuring device; ${|down\rangle }_{w}$ is the initial state of the weight which is positioned at some initial height we denote by down; and ${|e_{0}\rangle }_{e}$ is the initial state of the environment, which we assume does not interact at *t*_0_ with the particle or the weight (or the partition).

The initial energy state of the particle ${|x_{0}\rangle }_{p}$ is some superposition of energy eigenstates which depend on the width *a* of the box, where the amplitudes in ${|x_{0}\rangle }_{p}$ give the expectation value $\langle x_{0}\rangle$ for the energy of the particle. We assume for simplicity that the box is an infinite potential well so that all the energy eigenstates at the initial time have a node in the center of the box, and the quantum mechanical probability of finding the particle exactly in the center of the box is zero. In general, the quantum state of the particle ${|x_{0}\rangle }_{p}$ will be a superposition of energy eigenstates of the form: $\sin\frac{n\pi x}{a}e^{-\left(\frac{E_{n}}{\hbar }\right)t}$ with an eigenvalue $E_{n}=\frac{n^{2}h^{2}}{8Ma^{2}}$, where *n* is an even number and *M* is the mass of the particle. In standard quantum mechanics, such a superposition does not express ignorance about the energy eigenstates of the particle. It is the fine-grained complete energy state of the particle. In this sense one can say that such a superposition is the quantum mechanical analogue of a classical microstate.

${|S\rangle }_{m}$ is the standard ready state of the measuring device which is an eigenstate of the so-called pointer observable in this setup. For simplicity, we may take a spin-1 particle to represent the measuring device with ${|S\rangle }_{m}=|-1\rangle$ in the *z*-direction. The two other spin-1 eigenstates, |0〉 and |1〉 in the *z*-direction, correspond to the two possible outcomes of the measurement. Another alternative is to take the device to be a spin ½ particle, the pointer states in this case would be the up and down eigenstates of (say) the spin in the *z*-direction, and the initial ready state ${|S\rangle }_{m}$ would be a superposition of these two states with equal weights (e.g., either spin up or down in the *x*-direction).

At $t_{1}$ we insert a partition in the center of the box, where the wavefunction is zero, so that the expectation value for the energy remains completely unaltered. This is highly idealized, to be sure, creating many technical questions. For example, given the quantum mechanical uncertainty relations, one cannot insert the partition exactly at a point since in this case its momentum would be infinitely undetermined. To avoid this, one may assume that the partition and consequently also the particle are fairly massive, but that nonetheless they are kept at *t*_0_ in isolation from the environment. Alternatively, if there is a certain amount of decoherence, we may assume that the degrees of freedom in the environment are controllable in the subsequent stages of our experiment. Of course, assuming that the partition is massive, the idealization of zero width may also be problematic, and consequently the wavefunction may change when the partition is inserted. What we need is a way of inserting a partition such that it will not change the expectation values of the energy of the particle. In this sense we assume here that the effects of the above issues are negligible and we continue with our idealization. Again, we are not concerned here with the experiment’s feasibility, but rather with its consistency with thermodynamics.

We therefore take it that ideally at $t_{1}$ immediately after the insertion of the partition in the center of the box the quantum state of the setup becomes:

(2) $$|\Psi (1)\rangle ={|x_{1}\rangle }_{p}{|S\rangle }_{m}{|\mathrm{down}\rangle }_{w}{|e_{0}\rangle }_{e}$$

where

(3) $${|x_{1}\rangle }_{p}=\frac{1}{\sqrt{2}}({|L\rangle }_{p}+{|R\rangle }_{p})$$

and ${|L\rangle }_{p}$ and ${|R\rangle }_{p}$ are (in general) superpositions of the energy states of a particle in a box of width *a*/2, where for ${|L\rangle }_{p}$ the position *x* varies over the range [0, a/2] and for ${|R\rangle }_{p}$ *x* varies over the range [a/2, a], while the equal amplitudes are a consequence of our idealization that the partition is placed exactly in the center of the box. The expectation value of the energy of the particle is unaltered $\langle x_{0}\rangle =\langle x_{1}\rangle$ (On each side of the partition the superposition involves both odd and even eigenstates, but the width of the box is now *a*/2). Although the expectation value for the particle’s energy does not change in this interaction, the wavefunction of the particle now becomes a superposition of two components, as in (3). We assume that there are no other changes in the quantum state of the particle from *t*_0_ up to *t*_1_.

At $t_{2}$ a measurement of the coarse-grained position observable X of the particle is carried out, corresponding to whether the particle is located in the left- or right-hand side of the box. The quantum state immediately after the measurement, as described by the Schrödinger equation is:

(4) $$|\Psi (2)\rangle =\frac{1}{\sqrt{2}}({|L\rangle }_{p}{|0\rangle }_{m}+{|R\rangle }_{p}{|1\rangle }_{m}){|\mathrm{down}\rangle }_{w}{|e_{0}\rangle }_{e}$$

where the states ${|0\rangle }_{m}$ and ${|1\rangle }_{m}$ are the recording (pointer) states of the device, which are one-to-one correlated (respectively) with the locations of the particle given by the energy states ${|L\rangle }_{p}$ and ${|R\rangle }_{p}$. Notice that here we have assumed that the overall quantum state of the setup evolves unitarily and we do not apply the projection postulate. Additionally, we do not consider the question concerning the interpretation of this state, e.g., whether and in what sense the reduced state of the pointer, which is quantum mechanically mixed, records a determinate outcome of the measurement.

The measurement at time $t_{2}$ increases the von Neumann entropy of the device and of the particle, since the reduced quantum states of both are (improperly) mixed. However, this is irrelevant for the issue of Maxwell’s Demon in this setup since as we will see, the particle and the device will return to their separate pure states at time $t_{5}$, when their von Neumann entropy will decrease to its initial zero value. (For why the identification of von Neumann entropy with thermodynamic entropy fails, see our [57]. For an alternative understanding of the von Neumann entropy, see [6]).

At $t_{3}$ the partition is released, so that it becomes a piston. We assume that the piston is coupled to the weight in a way that is correlated with the outcome of the measurement, such that if the outcome is L the piston is free to move to the right, and the weight is lifted, and if the outcome is R, the piston is free to move to the left and the weight is equally lifted. In other words, there is a record of the outcome of the measurement in the motion of the piston, in the material of the weight, and in whatever connects them; but there is no record of the outcome in the height of the weight. Note that if the memory states of the device are not strictly orthogonal, there is a nonzero probability that the particle will be on the right side of the box and at the same time we shall release the piston from the right side, and in this case the weight clearly will not rise. However, nothing in quantum mechanics stands in the way of making this probability as small as we wish.

At $t_{4}$ the piston is pushed (to the left or to the right) by the particle. The quantum state of the setup at $t_{4}$ has the form:

(5) $$|\Psi (4)\rangle =\frac{1}{\sqrt{2}}\left({|L(w(t))\rangle }_{p}{|0\rangle }_{m}+{|R(u(t))\rangle }_{p}{|1\rangle }_{m}\right){|y(t)\rangle }_{w}{|e_{0}\rangle }_{e}$$

where the energy states |L(w(t))〉 and |R(u(t))〉 change with the changing width of the box, *w(t)* and *u(t)*, respectively. The expectation value of the particle’s energy ${\langle x(t)\rangle }_{p}$ is given by $\langle x(t)\rangle =\langle \frac{1}{2}\left(L(w(t))+R(u(t))\right)\rangle$ and decreases continuously over time in the interval from $t_{4}$ up to *t*_5._ The energy state of the weight ${|y(t)\rangle }_{w}$ changes accordingly, so that *y*(*t*) ranges from the down to the up position, and the expectation value of the energy of the weight increases accordingly. Conservation of energy, which is stated in terms of expectation values in quantum mechanics according to Ehrenfest’s theorem, implies that

(6) $${\langle x(t)\rangle }_{p}+{\langle y(t)\rangle }_{w}={\langle x_{0}\rangle }_{p}$$

Since the weight is positioned in a gravitational field, higher energy expectation values for the weight are coupled with higher positions of the weight. This means that the quantum-mechanical transfer of energy increases the probability that the weight will be found at higher positions upon measurement.

The Hamiltonian is such that during the expansion of the particle, as described by (5), the correlations between the memory states of the device and the energy states of the particle are gradually lost. The energy states ${|L(w(t))\rangle }_{p}$ and ${|R(u(t))\rangle }_{p}$ are such that at any time *t* the overall probability of finding the particle in the left- or right-hand side of the box is invariably ½. The conditional probability of finding the particle in the right- (left-) hand side, given that the device is in the state ${|0\rangle }_{m}$ (${|1\rangle }_{m}$) at $t_{3}$, is almost zero, but it increases with time and becomes ½ at $t_{5}$, since the correlations between the memory state of the device and the location of the particle are completely lost at $t_{5}$. At this stage, since the time evolution of the total state is reversible, there may be some records of the memory state of the device for each of the two components of the superposition. For example, there may be traces of the memory of the device in the state of the weight or of the pulleys, due to the difference in the motion of the piston to the right or to the left. We can use the memory in the pointer states of the device to erase these traces. When all this is done at $t_{5}$ the quantum state of the setup is given by:

(7) $$|\Psi (5)\rangle ={|x_{5}\rangle }_{p}\frac{1}{\sqrt{2}}({|0\rangle }_{m}+{|1\rangle }_{m}){|\mathrm{up}\rangle }_{w}{|e_{0}\rangle }_{e}$$

where ${|x_{5}\rangle }_{p}$ is the energy state of a particle in a one-dimensional box of width *a* with amplitudes which match the decrease in the expectation value of the energy, ${\langle x_{5}\rangle }_{p}={\langle x_{0}\rangle }_{p}-{\langle \mathrm{up}\rangle }_{w}$, and ${|up\rangle }_{w}$ corresponds to the up-state of the weight in which the expectation value of the energy is higher. The measuring device remains in the superposed state $\frac{1}{\sqrt{2}}({|0\rangle }_{m}+{|1\rangle }_{m})$, but the particle and the device are now decoupled (and disentangled). This means that at this stage there are no memories of the outcome of the measurement in the state of the device or in the state of any other system in the setup. If we take the device to be a spin ½ particle, then it is now back in the ready state. If the device is a spin-1 particle we need to evolve the state $\frac{1}{\sqrt{2}}({|0\rangle }_{m}+{|1\rangle }_{m})$ to its initial state ${|S\rangle }_{m}$. Since the device is no longer entangled with the particle, these two states are non-orthogonal pure states, and so there is a quantum-mechanical Hamiltonian that will do that.

To complete the cycle of operation we need to evolve the particle back to its initial energy state. We carry out this task by extracting energy from the environment *e*. For this purpose, we let the particle interact with *e* in such a way that the particle will certainly return to its initial energy state ${|x_{0}\rangle }_{p}$. Since we assume that the environment is initially in a pure state, possibly very complex but nevertheless a pure state rather than a mixture, there is a deterministic evolution that will yield precisely this energy transfer, no matter how complicated it may be. In this interaction, the expectation value of the energy of *e* decreases by the same amount as the expectation value of the energy of the particle (or ultimately the weight) increases, ${\langle e_{0}\rangle }_{e}-{\langle e_{1}\rangle }_{e}={\langle \mathrm{up}\rangle }_{w}-{\langle \mathrm{down}\rangle }_{w}$, as required by conservation of energy (where ${\langle e_{1}\rangle }_{e}$ is the expectation value of the environment’s energy after the interaction with the particle). (Strictly speaking, the energy of the environment is finite. For a discussion of the limitations of the idealization of an infinite heat bath, see our [7], Chapter 1)). Of course, the reverse evolution is also possible—namely that the particle will transfer energy to the environment—but we assume that the initial conditions in our setup match the first course of evolution. The expectation value of the weight’s energy is now greater than it was in its initial state.

The quantum state of the setup at the end of the process at *t*_6_ is this:

(8) $$|\Psi \left(6\right)\rangle =|x_{0}\rangle_{p}|{S\rangle }_{m}|{\mathrm{up}\rangle }_{w}|e_{1}\rangle_{e}$$

Therefore, the cycle is completed and the setup is ready to go once again.

What is a completion of an operation cycle? Once the cycle is completed, we do not want the environment and the weight to return to their initial state. Instead, the idea is that at the end of the cycle the situation will be such that the only change in the universe is that energy has been transferred from the environment to the weight as in state (8). Bennett and Szilard, for example, argue that completing the cycle of operation involves dissipation in the environment, and therefore the environment’s final state is a fortiori different from its initial state. For them, not only the state of the environment changes, but the entropy of the environment, increases. However, in our setup there is no reason to think that the transfer of energy from the environment to the weight results in an increase of entropy of the environment. Such increase does not follow from the equations of motion. (For example, as long as the interaction with the environment does not lead to decoherence (as we assume here for simplicity; see the last section), the von Neumann entropy does not change, since the environment evolves from one pure state to another). As we said in Section 1, one should not assume the Second Law in the context of the question of Maxwell’s Demon, since the Demon is meant to challenge the Second Law. We have thus demonstrated that a Maxwellian Demon is compatible with quantum mechanics without the projection postulate.

In our above discussion, we have phrased our argument in terms of entropy. There are different notions of entropy in the literature, for example, thermodynamic entropy (see [58]); Boltzmann’s entropy, Gibbs’s entropy (see e.g., [33,34,37]); and von Neumann’s entropy (see [57]). All these notions of entropy can be applied to our setup as long as they can be translated to quantum mechanics (for the notions of macrostates and entropy in quantum mechanics, see [1,2,3,4,5,6,59], and Appendix A). Our conclusion remains intact: we have a reversible quantum evolution of a closed system (including the environment), in which energy is extracted from the environment and transferred to the weight; the weight ends up in its up state; there are no traces of this process in the state of everything else; and the particle and everything else return to their initial states or to states of equal entropy. As long as the Second Law is not assumed from the outset, there is no reason to think that the entropy of any subsystem in the setup should increase at the end of each cycle.

## 3. A Demon in Quantum Mechanics with the Projection Postulate

Let us now consider our setup in the framework of standard quantum mechanics in von Neumann’s formulation with the projection postulate in measurement. As standardly understood, we take the projection postulate to express the so-called collapse of the quantum state: a pure-to pure (nonlinear and stochastic) state transition of a single setup in measurement. Applying the projection postulate at $t_{2}$ to the location measurement, the quantum state is either:

(9) $$|\Psi_{c}(2)\rangle ={|L\rangle }_{p}{|0\rangle }_{m}{|down\rangle }_{w}{|e_{0}\rangle }_{e}$$

or

(10) $$|\Psi_{c}(2)\rangle ={|R\rangle }_{p}{|1\rangle }_{m}{|down\rangle }_{w}{|e_{0}\rangle }_{e}$$

with probability ½, and at $t_{5}$ (before the interaction of the particle with *e*) it is either

(11) $$|\Psi_{c}(5)\rangle ={|x_{5}\rangle }_{p}{|0\rangle }_{m}{|up\rangle }_{w}{|e_{0}\rangle }_{e}$$

or

(12) $$|\Psi_{c}(5)\rangle ={|x_{5}\rangle }_{p}{|1\rangle }_{m}{|up\rangle }_{w}{|e_{0}\rangle }_{e}$$

The unitary Schrödinger equation cannot map the final states ${|0\rangle }_{m}$ and ${|1\rangle }_{m}$ of the device to ${|S\rangle }_{m}$. However, we can erase the memory of the device non-unitarily in a way suggested explicitly by von Neumann (see [56] Chapter 5). We can measure on the device an observable λ, which does not commute with the pointer observable. Subsequently, to bring the device back to its standard state, we measure the pointer observable (i.e., spin in the *z*-direction) on the device until the outcome corresponding to ${|S\rangle }_{m}$ is obtained, at which case the cycle is completed.

We assume that the second device required for the erasure of the memory by the above process is part of the environment. In general, the state of this second measuring device will be different from its initial state. However, the important point for the question of the Demon is that at the end of this process there is no memory in the state of this second device of the outcome, left or right, that has been registered earlier, and that there is no increase of entropy as a result of this second measurement. For a proof that a quantum measurement does not result in an increase of entropy in a collapse theory, see our [57].

By the above erasure of the memory of the device we have completed the thermodynamic cycle (as explained at the end of the previous section). This shows that a Maxwellian Demon is compatible also with quantum mechanics with the projection postulate.

## 4. The Connection between Measurement and Erasure

Often in the literature it is argued that the erasure of the memory is accompanied by dissipation, according to the Landauer–Bennett thesis. We have disproved this thesis in the classical case in our [7,18,19,20,21,22,57]. The physical details of erasure evolutions do not require any dissipation that would compensate for the decrease of entropy in our setup. Discussions of Maxwell’s Demon in the quantum mechanical context rely on the classical Landauer–Bennett thesis (see Earman and Norton [29] concerning Zurek [30]. There are no independent sound arguments in support of this thesis that are genuinely quantum mechanical.

The discussion of Maxwell’s Demon in the literature focuses on the notions of measurement and erasure. In the classical case, because of the determinism of the dynamics, these notions must be understood as referring to a macroscopic evolution, namely evolutions of sets of microstates (see our [7,18,19,20,21,22]). In quantum mechanics, as we saw in the previous sections, these two notions can be understood microscopically and are intertwined as we explain below.

Consider first measurement and erasure in standard quantum-mechanics without the projection postulate. In particular, let us describe only the transition of states of the measuring device, by tracing over the rest of the setup throughout the entire evolution. As we saw, the measurement and erasure evolutions of the measuring device *m* are given by the following sequence of states:

(13) $$|S\rangle \to \frac{1}{2}(|0\rangle \langle 0|+|1\rangle \langle 1|)\to \frac{1}{\sqrt{2}}(|0\rangle +|1\rangle )\to |S\rangle$$

where the first arrow stands for the measurement, the second for the erasure of the outcome, and the third for the return (or re-setting) of the device to the ready state (the third arrow is needed only if the device is the spin-1 particle). The measurement interaction (represented by the first arrow on the left-hand side of (13)) transforms the device from a pure to a mixed (i.e., improper) state; the erasure (represented by the arrow in the middle of the sequence) evolves the device from a quantum mechanically mixed (improper) state to a pure state, which is a superposition of the two memory states; and the resetting arrow (represented by the third arrow in the right-hand side of the sequence) transforms the device from this superposition to the initial standard state. The entire evolution described by (13) is possible, since it is brought about by the Schrödinger evolution of the global quantum state |Ψ(t)〉, which is time-reversible. Notice that although the von Neumann entropy increases by the first arrow, it decreases to zero by the second arrow, and therefore it has no effect on the total entropy change in the Demonic evolution. (See our [57] for a detailed argument explaining why the von Neumann entropy is not identical to thermodynamic entropy).

It might seem that the second arrow in (the middle of) (13) does not actually erase the outcome of the measurement, since the Schrödinger evolution is reversible. However, this impression is mistaken for the following reason. If one posits that the third arrow in evolution (13) does not bring about an erasure just because the dynamics is reversible, then by the same argument the determinism of the dynamics entails that the first arrow in (13) does not bring about a measurement with a definite outcome. Any account in which one obtains a determinate outcome at the end of the measurement, would also be an account in which the outcome is erased at the end of the erasure.

In standard quantum mechanics with the projection postulate in measurement, there are two possible evolutions of the quantum state of the measuring device (since the outcome of the measurement is stochastic), each with probability ½, as follows:

(14) $$\left|S\rangle \to \right|0\rangle \to \frac{1}{\sqrt{2}}(|0\rangle +|1\rangle )\to |S\rangle$$

or

(15) $$\left|S\rangle \to \right|1\rangle \to \frac{1}{\sqrt{2}}(|0\rangle +|1\rangle )\to |S\rangle$$

As before, the first arrow on the left-hand side represents the measurement, the second arrow represents the erasure and the third represents the return to the ready state (and again, the third arrow is needed only if the device is the spin-1 particle). As we saw in Section 3, the left or right location of the particle is measured by coupling the memory states |0〉 and |1〉 of the device to the states of the particle, and then collapsing the memory onto one of them. The memory of the outcome of the measurement is erased in the standard collapse theory by measuring the observable λ of the measuring device, which is quantum-mechanically incompatible with the pointer (or memory) observable. As we explain in Section 3 after this (second) measurement, which here plays the role of erasure, there is no record in the state of the second device of the outcome of the first location measurement, and there is no increase of entropy as a result of this second measurement.

In standard quantum mechanics (with and without the projection postulate), as we saw, the measurement of any observable that does not commute with the memory observable is an erasure, since after such a measurement it is impossible to retrodict the pre-erasure state of the memory system from the post-erasure state. In a collapse theory after a quantum erasure, one cannot even retrodict which observables had definite values in the past. In our setup, since λ is maximally incompatible with the pointer (or memory) observable, even if we assume that there is a memory of the identity of the pointer observable, one cannot retrodict even probabilistically which memory state, |0〉 or |1〉, and therefore which outcome of the measurement existed at $t_{2}$. This is quite unlike a classical erasure which can only be macroscopic, and therefore requires (what we have called in our 2012) a macroscopic blending dynamics. It is this sort of macroscopic dynamics that makes the recovery of macroscopic records (measurement outcomes, memories, etc.) impossible in the classical theory (see our [7,18,19,20,21,22]). In quantum mechanics, macroscopic blending is not needed just because measurements of observables that do not commute with the pointer observable (or the memory observable) described at the quantum mechanical microlevel are microscopic erasures.

In the context of classical statistical mechanics, we showed (see [7,18,19,20,21,22]) that macroscopic measurement and erasure need not increase thermodynamic entropy. Our analysis in the previous sections shows that the same conclusion carries over to standard quantum mechanics (with and without collapse) with respect to microscopic measurement and erasure (for the case of measurement and von Neumann entropy, see our [57]).

Finally, notice that the question of whether or not the evolution in (13) describes measurement and erasure per se, turns out to be immaterial to the issue at stake: what is important here is whether or not our cyclic evolution results in total entropy increase anywhere in the universe. As we saw, we have a reversible quantum evolution of a closed system (including the environment), in which energy is transferred from the environment to the weight; the weight ends up in its up state; at the end of each cycle all other subsystems return to their initial states or to states of equal entropy; and there are no further changes in entropy in the universe. This is why such an evolution is a Maxwellian Demon.

## 5. Maxwell’s Demon

In this paper we have shown that an entropy decreasing evolution, which includes measurement and erasure, is compatible with quantum mechanics. The entropy decrease in our setup, which is of a closed system (albeit highly idealized), violates the Second Law of thermodynamics. In the classical case there are no microscopic measurements and erasures. Therefore, the question of Maxwell’s Demon must be analyzed by introducing macrostates and macroscopic evolutions. In standard quantum mechanics, however, measurement and erasure can be constructed microscopically, as we have demonstrated above. In other words, in quantum mechanics there is no need to introduce quantum mechanical macrostates in order to consider the question of Maxwell’s Demon.

However, if one is interested in a macroscopic description of a Demonic evolution in quantum mechanics, it is important to note that the classical notions of macrostates and macroscopic preparations do not have trivial counterparts in quantum mechanics (on this issue, see [1,2,3,4,5,6]). In Appendix A we describe the quantum mechanical analogue of a classical macrostate. We believe that this notion of a quantum macrostate should be considered in the description of a macroscopic Demon in quantum mechanics. In order to carry out a macroscopic Demonic evolution analogous to the classical case, all we need is that the eigenvalue associated with the initial energy eigenstate of the particle ${|x_{0}\rangle }_{p}$ will be degenerate, and moreover that there will be more than one possible history of the particle that leads to that state by the same Hamiltonian (see Appendix A below). There is no no-go theorem in quantum mechanics which prevents the possibility of preparing a state of this kind. Since our Demonic evolution does not depend on any other details of the initial energy eigenstate of the particle, it applies to all the quantum states that belong to the degenerate energy eigenvalue. This means that typicality (or probabilistic) considerations that often come up in the attempts to account for the Second Law in statistical mechanics are irrelevant here. Therefore, it seems to us that as a matter of principle a genuine macroscopic Maxwellian Demon is consistent with quantum statistical mechanics.

This result implies that the Second Law of thermodynamics is not a universal theorem of quantum statistical mechanics. Our previous result of a classical Maxwellian Demon (see our [7,18,19,20,21]) implies that the Second Law is not a universal theorem of classical statistical mechanics. As we said, these results are not in conflict with the empirical evidence that supports Second Law behavior: the proof that Maxwell’s Demon is compatible with fundamental physics leaves open the possibility that both the entropy increase described by the Second Law and the entropy decrease described by Demonic evolutions are both compatible with fundamental physics; they may hold for different initial conditions of the universe. However, one implication of our result is that the Second Law cannot provide a lawlike basis for an account of the arrow of time; this account has to be sought elsewhere (for details about how this can be done, and why the Second Law cannot ground the direction of time, see our [60]).

Finally, in our construction of the Demonic setup above, we have assumed that the entire system consisting of the spin-½ particle, the measuring device, which is a spin-1 or spin-½ particle, the piston and the pulleys, which connect the piston to the weight, and everything else, are not subject to environmental decoherence. If some of these subsystems do undergo decoherence interactions with the environment, then of course we shall have to erase the records of the measurement outcome that might be present in the environment states. This can be done for example in a no-collapse theory by re-interfering the environment states in essentially the same way we described above with respect to the pointer states. Obviously, such re-interference would require complete control over many degrees of freedom in the environment, but there is no lawlike limitation in quantum mechanics (that does not depend on the Second Law) that prevents such re-interference. As we said, we are not interested here in practical limitations. Therefore, environmental decoherence does not impose a quantum mechanical lawlike limitation on our thought experiment.

## Appendix A. Quantum Macrostates

How can one prepare a macroscopic state according to quantum mechanics? We take it that the quantum mechanical counterparts of thermodynamic magnitudes are eigenvalues of certain observables. Suppose that we prepare a collection of systems by measuring on each of them the observable M, applying the projection postulate, and collecting only those with eigenvalue M_0_ (with the corresponding eigenstate or eigensubspace). Then (in the standard theory with the projection postulate, except in special cases described below) the resulting quantum state is known with complete accuracy: it is a pure state. Unlike the classical case of preparing a system by measuring a macrovariable on it, which leaves us with ignorance concerning the other aspects of the microstates, here the information about the quantum state is complete, not partial. No ignorance remains.

The only case (in standard quantum mechanics) where one can prepare a system with a certain eigenvalue and nevertheless remain with a set of quantum microstates compatible with the measured eigenvalue is the measurement of degenerate observables in special circumstances. In the general case, upon measurement of the observable A on some quantum state |ψ>, if the outcome is eigenvalue a_n_ with degree of degeneracy g_n_, then according to the projection postulate, the final state is a (normalized) superposition of all the g_n_ eigenvectors of A associated with a_n_. However, if the initial quantum state |ψ> is itself one of the eigenvectors associated with a_n_ then the final state will remain unchanged and will not become a superposition of all the eigenvectors of a_n_. This has the following consequence. Suppose that before A was measured, a non-degenerate observable B was measured non-selectively, and then a suitable Hamiltonian was applied on the resulting proper mixture, such that each eigenstates b_n_ of B in the mixture evolved to an eigenstate of the degenerate eigenvalue a_n_ of A. In this case, the state of affairs just prior to the measurement of A can be described in terms of a proper mixture of the eigenstates associated with a_n_ and this proper mixture will carry over to the state after the measurement of A, with the same (normalized) probability distribution. The result is that by the end of the measurement process we know that the quantum state is a definite pure quantum eigenstate of a_n_, but we do not know which it is—much like in a classical preparation. This is the only way to understand a preparation of macrostate, i.e., a set of microstates in standard quantum statistical mechanics.
